# Use of Real Patients and Patient-Simulation-Based Methodologies for Teaching Gastroenterology to Pre-Clinical Medical Students

**DOI:** 10.3390/healthcare6020061

**Published:** 2018-06-12

**Authors:** Joshua DeSipio, John Gaughan, Susan Perlis, Sangita Phadtare

**Affiliations:** 1 Department of Medicine, Gastroenterology/Liver Diseases Division, Cooper University Health, Camden, NJ 08103, USA; DeSipio-Joshua@CooperHealth.edu; 2 Cooper Research Institute, Cooper University Health, Camden, NJ 08103, USA; gaughan-john@CooperHealth.edu; 3 Office of Medical Education, Cooper Medical School of Rowan University, Camden, NJ 08103, USA; perliss@rowan.edu; 4 Department of Biomedical Sciences, Cooper Medical School of Rowan University, Camden, NJ 08103, USA

**Keywords:** gastroenterology, patient participation, patient simulation, active learning, peer teaching

## Abstract

In recent years, there has been an increasing focus on the need to integrate formal knowledge with clinical experience in the pre-clinical years since the initial years of medical education play an important role in shaping the attitudes of medical students towards medicine and support the development of clinical reasoning. In this study, we describe approaches that involve real patients and patient-simulation-based methodologies to teach gastroenterology to second year medical students. Our goals were to (i) demonstrate bio-psychosocial aspects of clinical practice, (ii) demonstrate commonality of gastrointestinal ailments, and (iii) help understand complex gastroenterology concepts. We used two main approaches including brief, pre-prepared questions and answers discussing with the patients in various sessions throughout the course and a two-hour session that included patient participation, patient simulation modalities with high fidelity mannequins, a lightening round of interactive cases, and a Patient Oriented Problem Solving (POPS) session. The approaches improved the effectiveness of the delivery of the content-heavy, fast-paced GI course and provided opportunities for the students to think about gastroenterology from both basic and clinical points of view. The approaches involved peer teaching, which supports knowledge acquisition and comprehension. Very positive feedback and overall engagement of students suggested that these approaches were well-received.

## 1. Introduction

The initial years of medical education play an important role in shaping the attitudes of medical students towards medicine and training them for their future role as a physician [1,2]. The Carnegie Foundation for the Advancement of Teaching report [3,4] emphasized the need to integrate formal knowledge with clinical experience in the learning environment. Numerous studies have shown the benefits of exposing students to patients prior to traditional clerkship rotations [5,6]. Benefits of early exposure to patient care include developing comfort with patients, developing efficient clinical skills, encouraging active learning, making learning more relevant, and reducing difficulty with the transition to clinical practice [7,8,9,10,11]. Therefore, for the past three decades, there has been a push to integrate clinical experiences into pre-clinical education. The growing consideration to provide some opportunities for integrating pre-clinical and clinical phases have resulted in implementing various types of vertically and horizontally integrated practical experiences into the early years of curricula in medical schools [12,13,14].

The Cooper School of Rowan University (CMSRU) received full LCME (Liaison Committee on Medical Education) accreditation in 2016 and graduated its third cohort of students in 2018. The pre-clinical curriculum integrates basic and clinical sciences and includes a mix of lectures, laboratories, and active learning activities. Our pre-clinical courses are comprised of approximately six hours with each Active Learning Group sessions (ALG), lectures, and afternoon lab sessions. Some courses also include team-based learning (TBL), jigsaws, or other interactive sessions. Attendance for the ALGs and some afternoon sessions is mandatory. Each ALG group consists of eight students and two faculty facilitators (one basic science and one clinician) and meets for two hours, three times per week. Students are given opportunities involving patient interaction such as the courses, Week on Wards (WOW), and an Ambulatory Clerkship at the Cooper Rowan Clinic. The Gastroenterology (GI) course is taught in the fall semester of the second year of medical school. It is a four-week course that integrates the biochemistry, pathophysiology, anatomy, histology, and embryology along with the signs and symptoms, diagnostic methods, and treatment modalities of GI, hepatic, and biliopancreatic diseases and nutrition.

The sheer volume of information that had to be presented to the students during the GI course made it a very fast-paced and somewhat daunting course. In response, we incorporated several methodologies in the course, which allow the students the opportunity to pause and reflect on the content being taught within the context of the big picture. Previously, we described the reception and efficacy of an interactive activity on nutritional pathology that was introduced in this course. The activity included discussion about various nutrition pathologies based on real-life cases, which made the exercise very clinically relevant to the students. The activity was very well received and helped enhance competency in nutrition for the students [15]. In this study, we described another activity that we used in this course, which involved real patients and patient-simulation-based methodologies. Our goals were to demonstrate bio-psychosocial aspects of clinical practice, demonstrate commonality of gastrointestinal aliments, and help students understand complex gastroenterology concepts.

## 2. Experimental Section

We used two main approaches: inclusion of (i) brief, pre-prepared question-and-answer sessions with patients in various sessions throughout the course and a two-hour session that included various elements such as patient participation, patient simulation modalities with high fidelity mannequins for demonstration of upper endoscopy and colonoscopy, a lightening round of interactive cases with questions and answers, and a new activity that is reminiscent of the Patient Oriented Problem Solving (POPS) methodology [15,16,17,18,19]. However, only half of this session was based on the POPS structure. It was collectively referred to as POPS (or POPS 2) for course scheduling and evaluation purposes and is described below as such.

### 2.1. Selection and Participation of Patients Throughout the GI Course

We selected six patients with each suffering from a different gastrointestinal disorder to participate in the course activities. The disorders were infection with *Helicobacter pylori*, gallstones, celiac disease, liver cirrhosis, ulcerative colitis, and irritable bowel syndrome. Most of the patients who participated were people who the students saw every day such as faculty and staff. The course directors met with the patients to learn about their disease, the symptoms they experienced, the treatments they were undergoing, the biopsychosocial aspects including the challenges they faced, and how these diseases influenced them and their families. We then prepared the key features from each of these discussions in the form of questions and answers and shared these with the respective participating patients for accuracy. We also included a note for the GI concept that we wanted to emphasize for each disease. This pre-preparation allowed the patient participants to be brief yet effective with respect to time and delivery in the specific class sessions. The patients participated in various sessions throughout the course. Two patients participated as content experts in ALGs with one of the course directors. Two patients participated during lectures and in the interactive session on diarrhea described below. The entire class met the patients who participated in the ALGs or in the diarrhea interactive session since attendance for these sessions is mandatory. Attendance at lectures is not mandatory. Since the lectures are recorded and the recordings are accessible to the entire class, those students who did not attend the lecture in person were able to watch the questions and answers sessions with the patients. Table 1 describes the patients who participated along with the sessions in which they participated and the main aspects of the respective GI diseases discussed.

### 2.2. Interactive Session on Diarrhea Cases

Diarrhea is caused by a variety of gastroenterology disorders including both with infectious and non-infectious etiology. Our main goals were clinical aspects and physiological manifestations of different GI diseases that cause diarrhea and to demonstrate to the students how endoscopy and colonoscopy can be used for the diagnosis of some of these diseases.

#### 2.2.1. Structure of the Diarrhea Session

The session was carried out in a large lecture hall with two faculty facilitators who are the course directors of the GI course. It was two-hours in duration and was divided in two major parts. The first part focused on non-infectious diarrhea and included a virtual upper endoscopy and colonoscopy demonstration, question-and-answer time with real patients, and a lightening round of upper endoscopy/colonoscopy-based interactive diarrhea cases. The gastroenterologist, serving as the GI co-course director, used simulation modalities such as standardized patients and high fidelity mannequins that realistically replicate the clinical environment to demonstrate how upper endoscopy and colonoscopy is carried out. The demonstration was also shown on the computer screens using screen-in screen projection. Students were able to see the gastroenterologist carrying out these procedures and, at the same time, were able to see what he observed in the simulated patient on the computer screen. The lecture hall in which this activity was carried out has computer screens on all the walls, which allows the students optimal viewing regardless of their location in the classroom. The gastroenterologist provided live commentary as the scope was travelling down the simulated patient’s GI tract. He also asked the students questions about different regions of the GI tract when the procedure progressed. This was followed by a question-and-answer session with real patient who suffered from irritable bowel syndrome (IBS). The last component of the demonstration was a lightening round in which four case stems were displayed individually with their endoscopy/colonoscopy presentations and discussions followed about what the underlying diseases were. The diseases represented non-infectious GI diseases with diarrhea as one of the main symptoms including IBS, Crohns, Celiac disease, and VIPoma. The second part of the session included a group activity and a post-quiz. The students learned about four infectious diseases causing diarrhea (Enterotoxigenic *Escherichia coli*, *Giardia lamblia*, *Vibrio cholerae*, *Clostridium difficile*) during the group activity, which was based on the Patient Oriented Problem Solving (POPS) methodology [15,16,17,20]. Details of the POPS session are described below. Except for Celiac disease, the diarrhea session introduced the students to all of these diseases for the first time in the GI course. Discussions of other aspects of these diseases occurred in later lectures. The timeline of this entire session is below.

- Demonstration of endoscopy and colonoscopy using simulation modalities (25 min)
- Questions and answers session with real patient suffering from IBS (20 min)
- Lightening round of four, non-infectious GI diseases with diarrhea (15 min)
- POPS session with four infectious diarrhea cases and post-quiz (60 min)

#### 2.2.2. Preparation Required before the Diarrhea Session

The students were given a brief description of the main goals and format of the two-hour session in the course introduction lecture. Detailed instructions for the session were posted in advance on the course website. The only preparation required was for the second part of the session, which entails the POPS activity. The students were asked to read one page containing four stems that described clinical presentation of the four GI POPS cases, but were not required to study these cases or carry out research about these. Additionally, 80 students were randomly assigned to 20 groups of four students each. Each student was given a group number and a color (blue/green/purple/yellow) that corresponded to one of the four infectious diarrhea cases. They were asked to sit in their group when they arrived for the session.

#### 2.2.3. POPS Cases and Quiz

Each student within the group was assigned to learn about one of the four infectious diarrhea cases. Each case was assigned a color such as green (Enterotoxigenic *Escherichia coli*), purple (*Giardia lamblia*), yellow (*Vibrio cholerae*), or purple (*Clostridium difficile*). Having colored sheets was very useful for fast distribution during the session. During this part of the session, the students were given one colored page containing the information about their stem with the clinical presentation and patient history as well as laboratory data for one of the four diseases. The underlying cause, physiology and treatment options for each disease was presented in question-and-answer form to facilitate thought-provoking discussions among students. Important concepts were underlined or bolded. Highlights of each case are given in Table 2. All of these were actual cases modified to suit the purpose of the activity. The students were given approximately 10 min for reading their respective case materials. Each student then presented highlights of his/her material to the other three students in the group. They were allowed to carry out additional research on any aspects they wished to know more about and also allowed to ask the faculty facilitators for help if needed. After each of the four students at each table finished discussing their cases, the students took a post-quiz as a group, which contributed towards 3% of the final course grade.

Since the materials covered in the diarrhea session was not taught before this session, we felt that it was not appropriate to have the students take a pre-quiz. They were only asked to take a post-quiz. The quiz consisted of eight, USMLE Step 1-style questions. An example of one question is shown in Table 3.

### 2.3. Outcome Measures of the Approaches Used

#### 2.3.1. Evaluation of Reception of the Approaches

We assessed the efficacy of the approaches described here using three modes of evaluation by the students. The CMSRU Office of Medical Education (OME) collects students’ overall evaluation of the course as well as the evaluation of each of the course sessions (lectures, ALGs, TBLS, POPS, jigsaws, and labs) as a standard practice. These evaluations are collected anonymously following the school policies and are distributed to the course directors and participating respective faculty in an aggregate manner. We also carried out an additional evaluation of the approaches described here via a paper survey. The standard, electronic evaluation of the diarrhea session carried out by the CMSRU OME is designated here as Evaluation A. The additional paper survey collected after the completion of the diarrhea session is designated as Evaluation B. The comments pulled from the two questions asked in the standard, electronic overall course evaluation as described below are designated as Evaluation C.

##### Evaluation A

The students are asked to evaluate each session in a course. Since the students have to complete a large number of surveys, the school has adapted a policy that reduces the number of surveys each student has to complete in a course. According to this policy, about half of the class is asked to evaluate each interactive session. Therefore, 37 students completed the survey for the diarrhea POPS session. We have observed that the outcomes of these surveys are representative of the perception of the entire class. The standard evaluation form that is used for any interactive session (e.g., TBLs, jigsaws, POPS) includes eleven Likert scale questions: (i) the objectives of the session were clear, (ii) the session was well organized, (iii) the session was relevant to my education, (iv) the content helped me meet session objectives, (v) the session content was related to course objectives, (vi) the session stimulated me to want to learn more about the subject, (vii) the faculty maintained my interest, (viii) the faculty demonstrated appropriate knowledge, (ix) the faculty explained the material clearly, (x) the faculty used questions and student participation effectively, and (xi) the faculty demonstrated professionalism. Students are also asked to provide qualitative comments on the session.

##### Evaluation B

As mentioned above, the evaluation form A is a common form used for any interactive session in any given course by the CMSRU OME. To maintain consistency for evaluation across various courses in the curriculum, the evaluation forms are not modified for individual courses/sessions. We, therefore, decided to include an additional evaluation in the form of a paper survey to assess the reception of the inclusion of patients throughout the GI course along with the inclusion of the patient simulation in the diarrhea session. The authors have used additional paper surveys to assess efficacy of individual interactive sessions before [15,16]. The paper survey was distributed to the students at the end of the diarrhea session. Since this session is scheduled towards the end of the GI course, it provided the last opportunity to have the entire class in one place. The paper survey contained three Likert style questions and one open-ended question. The Likert scale questions were: (i) Was the inclusion of GI patient(s) during this exercise and throughout the GI course informative with respect to the biopsychosocial aspects of the GI disorders? (ii) Did the inclusion of GI patient(s) during this exercise and throughout the GI course enhance your empathy towards them? and (iii) Did you find working with your peers and the interactive nature of this activity conducive to learning about the GI system? The fourth question asked for any comment on the activity with respect to its structure and usefulness and inclusion of patient simulation elements. We received prior approval from the Rowan Institutional Review Board (IRB) (project ID: Pro2016001014) to conduct the paper surveys and also to use the data from the surveys and quizzes for publication. The anonymous paper surveys were administered as per the guidelines set by Rowan Institutional Review Board and did not contain personal identification markers. The students were informed that the evaluation of this activity was voluntary and anonymous and did not influence their grades.

##### Evaluation C

As mentioned above, in addition to the evaluation of each of the course sessions, the CMSRU OME collects students’ overall evaluation of the course as a routine practice. The entire class is asked to complete the overall evaluation for the course. Two of the several questions included in this form are: (i) Were there any sessions that were particularly outstanding? and (ii) What was the most vivid/thought-provoking, useful, or otherwise memorable information they learned in the course? We used comments received for these two questions as evaluation C for this study.

#### 2.3.2. Evaluation of Efficacy of the POPS Activity via Student Performance in Post-Quiz

In order to do well in the post-quiz, each student in a group needed to be a responsible member of his/her group and effectively present the respective material to his/her teammates. The students also needed to carefully read and synthesize the information and understand the underlying concepts. This was especially imperative since this was the first time the diarrhea cases were presented to them. The efficacy of the session as evidenced by post-quiz scores demonstrated that the session was highly effective as only one group got one question wrong. The concepts were further reviewed and consolidated in a one-hour lecture the following week.

## 3. Results

### 3.1. Evaluation of Reception of the Approaches

The students’ reception of these approaches was very positive. All the comments received for the open-ended questions in evaluations A, B, and C are given collectively in Table 4. The origin of each comment is given as a superscript at the end as ^A^, ^B^, or ^C^. The thematic analysis of the comments suggests that the main aspects liked by the students were team work, peer learning, helpful for understanding, and enjoyable, engaging involvement of patients including humanizing medicine, retention of concepts, and patient simulation. There were several additional positive comments on the inclusion of the patients, patient simulation, and also about this POPS in the various other evaluations collected for the GI course. It is also interesting to note that there were no negative comments about these approaches in any of the evaluations collected, which suggests their very positive reception in general.

**Figure 1:**
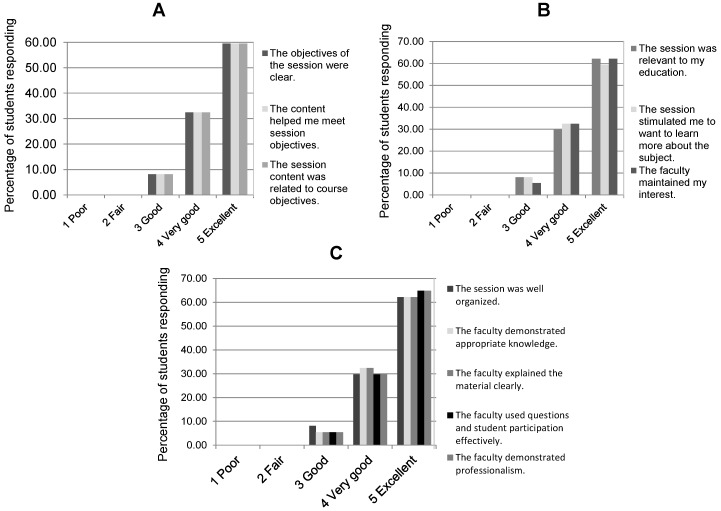
Quantitative representation of the student evaluation data of the interactive session with respect to (**A**) usefulness of the learning objectives, (**B**) relevancy and interest, and (**C**) structure and execution. The students evaluated the activity, which contained eleven Likert scale questions (five choices are shown) regarding these aspects.

Results of the eleven Likert scale questions in the Evaluation A are presented in Figure 1.

The results of the responses received for the three Likert scale questions in evaluation B are presented in Table 5. Out of the 80 students who participated in the interactive session, 77 chose to complete the paper surveys. The electronic data in Evaluation form A was provided to us in an aggregate form. However, the paper surveys allowed tabulation of the data for each response. Therefore, we were able to calculate Cronbach’s Alpha for evaluating the involvement of patients in the course. The Cronbach’s Alpha was 0.7, which suggests that there is a strong internal consistency and reliability of the items in the survey measurement [21].

### 3.2. Student Performance in the Post-POPS Session Quiz

There were a total of 20 groups with 80 students in groups of four who took the post-quiz. 19 groups answered all of the eight questions in the post-quiz correctly while one group got one question wrong. This was remarkable since the students were exposed to new material for the first time during this session. The concepts discussed in the POPS session were briefly reviewed and consolidated in lectures following the session. The students did very well on the questions based on these topics in the final course exam. 

## 4. Discussion

Early clinical exposure (ECE) integrates the knowledge of basic and clinical sciences and the psychosocial aspects of the medical practice [22]. This approach helps to facilitate the transition from pre-clinical years to clinical years. It also enhances students’ motivation and supports their appreciation of the relevance of basic sciences in clinical practice [2,8,11,23]. A study based on reflective essays written by fourth-year medical students [24] showed the influence of two pre-clinical education aspects that students noted on their conceptions of altruism, compassion, and respect. They perceived that the classes that allowed them to consider biopsychosocial aspects of patients’ lives before implementing a course of action in their medical care had a positive influence while the academic environment or culture itself that emphasizes their performance on exams to be anti-altruistic. Different types of early patient experiences may provide unique learning outcomes and acculturation for preclinical medical students [8]. An education model proposed by Dornan and colleagues [25] emphasized the importance of supported participation in clinical learning and suggested that the educational environment that supports and challenges learners can increase their participation within it. This, in turn, helps them develop additional competencies such as study strategies and clinical skills, which motivates them and may also help students to develop confidence and professional identity. This model emphasized that participation is central to student learning and that learning is shaped by human interactions.

High-fidelity patient simulation has been increasingly used as a teaching modality for health professional students. Life-size manikins that mimic real patients are used to simulate normal and disease conditions. These have been mainly used for residents or medical students in their clinical years. However, some studies indicate that patient simulators have been successfully used for first-year medical students coupled with problem-based learning to reinforce curricular concepts while bringing the cases to life [26,27,28,29,30,31].

We had given careful consideration in selecting patients for participation in the GI course and the aspects we wanted to emphasize for each disease. Important points include prior preparation of question-and-answer-based, patient-doctor scripts allowing the information to be delivered in a very succinct and time-efficient manner. Most of these patients in our learning activity were people the students encountered at the school every day. This emphasized the point about how common gastroenterology diseases are. Additionally, since the students knew these ‘patients,’ they were very comfortable asking them questions. The patients were chosen to emphasize specific aspects of GI disease. For example, we were able to discuss the genetics of and cross-relationship between different autoimmune disorders using the celiac disease patient while the gallstone patient was able to demonstrate how the gallstone disease can switch between acute and chronic phases, which is an aspect that the students usually find difficult to grasp. Patient participation encounters were brief given the time-constraints of the course, which were dispersed in different sessions throughout the course and aligned with the materials taught during respective weeks and ensured that all the students were exposed to the patient interviews. Each patient interview included the challenges they face living with a GI disease, which humanized the experience for the students.

Important considerations for the two-hour diarrhea session include the patient simulation that was designed to demonstrate the healthy state versus the disease state of the GI system using scoping methodologies. Students were asked questions during this demonstration about which area of the GI tract they were observing along with comments on what they expect to see in that organ for a particular disease. This created further engagement in the demonstration and encouraged them to think about and build on the GI concepts they had learned in the course. The interactive lightening round cases and questions further consolidated the diagnostic role of endoscopy and colonoscopy in various GI disorders. The POPS cases were designed to promote team work and peer teaching to learn new materials in a time-efficient manner, which is consistent with the notion that peer teaching enhances comprehension since it depends on sharing both cognitive and social congruence [32,33,34]. The complexity and length of all the case materials were comparable and appropriate for the duration of the session along with the highlighting of important points to reduce the need for additional guidance during the session and to ensure consistency for all groups. The session was facilitated in a single large room with two faculty members, which eliminated the need for additional faculty time or multiple rooms. The session did not require much prior preparation from the students, which suited the fast pace and time constraints of this very content-heavy course. Being able to take the post-quiz in a group further enforced reliance on peers and reduced the stress of test-taking, which allowed the students to focus on thinking critically about the concepts rather than worrying about performance on the quizzes. This, in turn, allowed them to enjoy the activity as an education experience. The quiz questions required the students to know the underlying mechanisms and employ higher-level thinking to apply that knowledge to clinical scenarios.

As evidenced by the very positive student reception of these approaches and their detailed comments, our goals for these approaches were achieved. Students stated that inclusion of patients in the course allowed them to put a ‘face’ on the disease and gave a practical connection that helped them to visualize it. They experienced patients in real time with respect to their needs and challenges, which provided them with better insight into the pathophysiology of the case at hand. These approaches gave them an opportunity to ask questions, which consolidated their learning and enhanced their connection to the patients. Since the interactions were designed in the form of questions and answers with the gastroenterologist course director of the GI course, the students had the experience of how a physician might interact with a patient and create a doctor-patient bond rather than solely looking at the patient as a case to treat. This may contribute to them becoming more invested in their future patients’ welfare. They also stated that the interactive approaches helped make the concepts more memorable and easier to understand.

One limitation of this study is that we examined a cohort of medical students in one medical school. However, benefits of early patient exposure have been reported in various healthcare disciplines such as nurse anesthesia students, dental students, and optometry students [7,35,36]. Although our evaluation strategies primarily focus on student satisfaction with the learning experience and post-quiz, these prior studies suggest that early experiences help students understand and align with patient and community perspectives and generate evidence for perceived and actual benefits of preclinical experience in front-loaded clinical programs. Another limitation of this study is that, while the POPS part of the activity works well with small groups (4–5 students), it limits how many diseases can be taught in a specific learning session. Assigning more than one case per student may not work well since it will require the students learn a large volume of material and teach it to their peers in a short amount of time.

## 5. Conclusions

We designed and implemented several approaches that involved real patients and patient simulation in the M2 GI course to stimulate student interest in gastroenterology, help them understand complex GI concepts, and demonstrate the bio-psychosocial aspects of the clinical practice. The approaches improved the effectiveness of the delivery of the GI course in that these learning activities provided opportunities for the students to think about gastroenterology from both basic and clinical perspectives. The POPS activity was based on peer teaching that involves cognitive development as well as social collegiality and, therefore, plays an important role in enhancing knowledge acquisition and comprehension. The excellent overall class performance in the post-quiz demonstrated that the students were able to learn and effectively apply new concepts in a short amount of time. Extremely positive feedback about the experience and the overall engagement of students suggested that these approaches were well received, which encourages us to implement them in other courses in our curriculum.

## Figures and Tables

**Table 1:** Highlights of the patient participation.

GI Disease	Session	Highlighted Points of Discussion
*Helicobacter pylori* infection	ALG * about *H. pylori*, acid-peptic disease	Recurrent infection due to antibiotic resistance of *H. pylori*, importance of confirmation of eradication, symptoms, diagnostic methods, treatment received, understanding the challenges patients face with the side effects of the treatment for this disease, life style changes.
*Gallstone*	Lecture on biliary diseases	Symptoms, demographic attributes of vulnerable population, treatment (type of surgery), pathophysiology of changing from chronic to acute to chronic disease states, life style changes, emphasis on talking to gallstone patients about seeking help in a timely manner.
*Liver cirrhosis*	Lecture on non-viral hepatitis	Underlying cause-alcoholism-pathophysiology, symptoms, treatment received, effect on overall quality of life, challenges faced due to dietary restrictions, importance of family support.
*Celiac disease*	Interactive session on diarrhea	Symptoms, how disease was diagnosed, treatment, dietary changes, overall effect on quality of life, family history of the disease-role of genetics, relationship to other autoimmune diseases (e.g., Graves Disease), inadequacy of screening methods.
*Irritable bowel syndrome*	Interactive session on diarrhea	Symptoms, demographic attributes of vulnerable population, triggers, challenges involved in diagnosis particularly colonoscopy, treatment options, dietary modifications, life style changes.
*Ulcerative colitis*	ALG * about inflammatory bowel syndrome	Symptoms, diagnosis, pathophysiology, challenges and life style changes after surgeries (colectomy and ileostomy), advice to the students as future physicians about how talking to these patients will be beneficial.

* ALG: Active Learning Group.

**Table 2:** Highlights of the infectious diarrhea cases used in the session.

Deficiency	Case	Major Points Discussed
*Clostridium difficile*	Patient acquired *C. difficile* infection after treatment with clindamycin at the hospital for a different issue.	Diagnosis, risk factors for *C. difficile infection,* microbiology, and pathophysiology of *C. difficile-mechanism of action of exotoxins.*
Enterotoxigenic *Escherichia coli* (ETEC)	Large scale outbreak of diarrhea in an office picnic.	Differentiating the different types of *E. coli* that cause diarrhea, symptoms, pathophysiology-mechanism of heat-labile and heat-stable *E. coli* toxins, treatments.
*Giardia lamblia*	*Giardia lamblia* infection after pancreas-kidney transplantation.	Epidemiology of *Giardia* infections including the unusual mode of acquiring infection, diagnosis- biopsy presentation, symptoms and treatment, life cycle of *Giardia*.
*Vibrio cholerae*	A patient with watery diarrhea.	Symptoms, diagnosis, pathophysiology-mechanism of action of enterotoxin, reasoning behind treatment options.

**Table 3:** Example of a quiz question.

Question. A 50-year-old man has to undergo a dental procedure. He has an artificial heart valve. Therefore, he was given clindamycin to prevent bacterial endocarditis. A week later, he has discomfort in the lower abdomen and develops watery diarrhea. Which one of the following explains the mechanism of action of toxin that is responsible for his condition?
Activation of enterocyte cyclic GMP (guanosine monophosphate) Stimulation of the vagus nerve in the abdominal viscera Inactivation of regulatory pathways mediated by Rho family proteins Activation of Gsα through an ADP (adenosine diphosphate)—ribosylation reaction

**Table 4:** Students comments received.

**Team Work**
1. I think these sessions are really helpful in learning the material. By reading the article and teaching it to our fellow team members, I feel we are learning the information even better than if we read it alone on our own. You need to really know something before you can teach it to someone else. Additionally, the cases that present the different material help me to remember the information even more. During the exam, I found myself thinking back and saying “Oh that was the blue case in the pops session.” It really helped with recall of the information. ^A^
2. I think the POPS really helps the students more than a normal TBL session. Learning from peers helps to make the material easier to understand and remember. ^B^
3. The POP sessions because of the method of learning it involved and required. ^C^
4. The POPS sessions were very helpful to discuss topics with other students. I was able to remember the material better through these discussions. ^C^
**Helpful for Understanding**
5. These are always excellent! I learn so much! ^A^
6. SO HELPFUL. ^A^
7. Helpful to have this before the diarrhea lecture. ^A^
8. Interactive sessions were helpful. However, visual simulation was not the greatest quality in MRP small screens. Perhaps (Faculty name) can show real pictures/videos from actual cases. ^A^
9. Great and helpful session. ^A^
10. POPS are very helpful! ^B^
11. POPS are great for learning. ^B^
12. I thought that the lightening round questions were super helpful!! ^B^
13. All aspects of the activity are very effective and a great aspect to the course that I enjoy. ^B^
14. Well-orchestrated and effective learning environment. ^B^
**Enjoyable, Engaging**
15. Perfect! ^A^
16. Very helpful and enjoyable! ^A^
17. Thank you Dr. (Faculty name) for consistently engaging us in the learning process! We appreciate the work you put into pulling this together! ^A^
18. Keep doing a great job. ^A^
19. Great course. ^B^
20. I love Pop! ^B^
21. Well done course. ^B^
22. These are awesome. Pop never looked or sounded so good. ^B^
23. This is the best Pop I’ve ever had!! I hope every pop is like this pop! ^B^
24. Love these sessions. ^B^
25. Very helpful. ^B^
26. Replaces TBLs with this. ^B^
27. I liked. ^B^
28. It is great!! ^B^
29. I love (faculty name)’s POPS. ^B^
30. It was great. ^B^
31. Love it!!! ^B^
32. Great! ^B^
33. Please expand this to all blocks! ^B^
34. Good activity. ^B^
35. I love this format!!! ^B^
36. POPs are much better than TBLs. ^B^
37. I really enjoyed the POPS sessions. ^C^
38. POPs session number 2. ^C^
39. Really enjoyed the POPS 2 session! ^C^
40. I loved the pop sessions. ^C^
41. The POPS were great. ^C^
42. Pops. ^C^
**Involvement of Patients: Humanizing Medicine, Retention of Concepts**
43. It brings perspective to the concepts we learn and puts a face to a story. ^B^
44. Really helped being able to walk through different clinical scenarios and having the patients helps humanize it. ^B^
45. Nice to get the patient perspectives. ^B^
46. I LOVE it—I strongly believe this should happen in all courses in order to increase our understanding of the patients. We are treating people, not disease! I think it would also go a long way toward promoting empathy. ^B^
47. I really appreciated the real patient experiences. ^B^
48. I think every course should bring in patients. Not only does it enhance our clinical understanding, but it also helps us. ^B^
49. POPs are awesome! Great experience. Patient experiences are vital! Include them in (another course name)! ^B^
50. Loved the Alcoholic liver patient’s story, the clinical correlation, and the human aspect of the session. It is so important that med students learn NOT to blame patients even if they are somewhat responsible for their disease course. ^B^
51. The addition of patients is a good idea. ^B^
52. It would be helpful to have good/bad interactions the patients experienced with their doctors plus the frustrations of suffering from symptoms while waiting to determine diagnosis. ^B^
53. I loved hearing from the patients. I wish every course did this. ^B^
54. Helps to understand how patients react to illness. Gives me a 10000 feet view of the disease process. ^B^
55. I LOVED having the patients come in and share their experiences. I thought that was a really powerful part of the course and I hope they continue to do this. ^C^
56. Enjoy the patient stories and POP sessions. ^C^
57. I thought the real life cases were a great way to integrate the course with the clinic. ^C^
58. I loved the POPs sessions and the personal stories from patients. They both helped solidify a lot of information.
59. Hearing patient stories. ^C^
60. I enjoyed having the real patients coming in and telling their stories. ^C^
61. I also enjoyed hearing from the patients. ^C^
62. I loved the session with the patient who had liver cirrhosis due to chronic alcohol consumption. It was a very “human” perspective and story. It really touched me and helped me apply the information I was learning in the lecture environment to the real life experience of patients. ^C^
63. The guests that were brought in throughout the block were really thoughtful and well related to the topics of the day. ^C^
64. I loved when (Faculty name) brought in patients to talk to us about their experiences. Very thoughtful! ^C^
65. I really liked the personal patient stories. Additionally, it was easier to remember the material from the POPs session.
66. Physical exam and patient experiences. ^C^
67. Personally enjoyed the patient profiles the most. I think that adds a much needed piece to the puzzle that is often lost during the first and second year. Reading an ALG case about a fictional person is one thing but being presented with actual people reminds you that these diseases affect actual people. ^C^
68. Patient stories. ^C^
69. Hearing stories from the real-life patients was very, very helpful and insightful. ^C^
70. All of the stories told to help give context to the information were very useful. ^C^
71. I really enjoyed having the volunteer patients come and talk with us. It gave a different perspective on “cases” that we were learning about and helped us to have a better appreciation for the topics we were discussing. ^C^
72. Meeting patients and having them share the way these illnesses affect their everyday life. Sometimes it’s easy to skip over that or not even think about it during lectures. ^C^
73. Honestly, the most thought-provoking information came from the visiting patients. ^C^
74. It was very interesting to learn how GI issues are so common in society and how greatly they can affect a patient’s quality of life. ^C^
75. I LOVED THE USE OF PATIENT STORY-TELLING. It was incredibly meaningful and enriching to hear the patient experiences and it incorporated psychosocial elements into our curriculum. This was an excellent idea. Please continue. ^C^
76. Including the patient sessions. ^C^
77. Please have more POPS sessions. I learn so much more from this active learning session than from TBL quizzes. This is a great component of the curriculum. It really helps us retain information. ^A^
78. The patients added a face to the diseases and disorders. This also makes it easier to remember because it is a story as opposed to a lecture. ^B^
79. Hearing patient stories helps solidify the information we are learning. ^B^
**Patient Simulation**
80. The demo was informative and I enjoyed the patient story. ^A^
81. One of my favorites of the block! Seeing the colonoscopy was awesome. ^A^
82. This was a great session! The endoscopy/colonoscopy demonstration with cases was interesting. I really enjoyed learning about IBS from (patient name) as well. ^B^
83. I really enjoyed seeing how a colonoscopy is done. ^B^
84. Really liked the endoscopy/colonoscopy. ^B^
85. All the times we had patients come to class/pops—I’ll never forget! Additionally, the virtual colonoscopy was so cool. ^C^
86. Learning about the different causes of diarrhea and the poop visual has really stood out. ^C^

^A^ Comments about diarrhea session (Patient-Oriented-Problem-Solving; POPS 2) (question in the standard electronic survey). ^B^ Any comment on the activity with respect to POPS 2 structure and usefulness and inclusion of patient simulation elements (question in the paper survey). ^C^ Were there any sessions that were particularly outstanding? What’s the most vivid/ thought-provoking, useful, or otherwise memorable information you’ve learned so far in this class? (questions in the electronic overall course evaluation survey).

**Table 5:** Quantitative presentation of students’ responses to the involvement of patients in the gastroenterology course.

Item	Percent Responding					
	Strongly Disagree	Disagree	Neutral	Agree	Stronly Agree	Mean Score
Likert Scale	1	2	3	4	5	
Informative	0.00	1.30	5.19	10.39	83.12	4.8
Enhance empathy	1.30	0.00	2.60	15.58	80.52	4.7
Conducive to learning	0.00	0.00	1.30	28.57	70.13	4.7
